# Activated Notch signaling augments cell growth in hepatocellular carcinoma via up-regulating the nuclear receptor NR4A2

**DOI:** 10.18632/oncotarget.15576

**Published:** 2017-02-21

**Authors:** Bo Zhu, Lichun Sun, Wei Luo, Min Li, David H. Coy, Long Yu, Wenbo Yu

**Affiliations:** ^1^ State Key Laboratory of Genetic Engineering, Collaborative Innovation Center for Genetics and Development, School of Life Sciences, Fudan University, Shanghai 200433, China; ^2^ Hunan Province Cooperative Innovation Center for Molecular Target New Drug Study, Institute of Pharmacy and Pharmacology, University of South China, Hengyang 421001, China; ^3^ Department of Orthopedics, Xiangya Hospital, Central South University, Changsha, Hunan 410008, China; ^4^ Department of Medicine, School of Medicine, Tulane Health Sciences Center, New Orleans, LA 70112-2699, USA; ^5^ Department of Radiation Oncology, Ruijin Hospital, Shanghai Jiaotong University School of Medicine, Shanghai 200025, China

**Keywords:** hepatocellular carcinoma, Notch signaling, Notch1, nuclear receptor, NR4A2

## Abstract

Hepatocellular carcinoma (HCC) is one of the most malignant cancers. Conventional therapies are limited due to the human liver being such a unique organ and easily showing side-effects. The unclear molecular mechanisms are tough challenges for scientists searching for new and effective anti-HCC targeting drugs. We identified that the nuclear receptor NR4A2 is a novel oncogene in HCC progression. In this study, we show that NR4A2 and the notch recceptor Notch1 were expressed highly in primary HCC tissues and immortal HCC cells by using qPCR, western blot and immuno-histochemistry assays. Both genes were observed to stimulate HCC cell proliferation, anti-apoptosis and cell cycle arrest by using cell proliferation assays and FACS assays. We also observed that the four notch receptor subtypes (Notch1-4) displayed different effects on HCC cell growth. The over-expression of Notch1 by transiently transfecting the intracellular domain of Notch1 (ICN1, Notch1 active form) increased the expression of NR4A2, with the knockdown of Notch1 decreasing NR4A2. This indicates that NR4A2 is one of the Notch-mediated downstream genes. Moreover, both NR4A2 and Notch1 suppressed the expression of tumor suppressors p21 and p63. These findings support that Notch1/NR4A2 co-regulate HCC cell functions by playing oncogenic roles and regulating the associated downstream signaling pathways. Novel Notch1/NR4A2-mediated oncogenic signaling may provide us a great opportunity for anti-HCC drug development.

## INTRODUCTION

Hepatocellular carcinoma (HCC) is the most prevalent primary liver cancer, with high mortality worldwide and a low five-year relative survival rate [1]. Traditional therapeutics are limited due to the toxic side-effects on this unique organ. Currently, the kinase inhibitor Sorafenib is the only FDA-approved drug for the treatment of advanced HCCs [2]. There is an urgent need to develop more effective anti-HCC drugs. However, the HCC pathogenic molecular mechanisms remain unclear [3, 4] although HCC is associated strongly with hepatitis, particularly the viral infections involved with the hepatitis B and C viruses [5, 6]. Thus, it is a tough challenge to develop novel anti-HCC drugs. Activated Notch signaling is believed to be involved in HCC pathogenesis, and more importantly, to be critical in HCC progression [7, 8]. It may represent a potential therapeutic approach in targeting this signaling pathway [9].

Notch signaling has been known to be highly conserved [9, 10] and is critical in the determination of a cell's fate [9]. Notch signaling is involved in cancer progression by acting as either a tumor suppressor in some types of cancers [9, 11–13] or as an oncogene in others [8, 9, 13, 14]. In HCC tissues and immortal cancer cells, Notch signaling is activated aberrantly [9, 15]. For instance, Notch1 and Notch3 were detected in 60% and 78% of 60 HCC patients, respectively [16]. Another report showed that Notch1 was observed to be activated in over 81% of human HCC specimens compared to the surrounding normal tissues [17], and also, Notch3 and Notch4 were expressed highly in human HCC tissues, with Notch3 present in 78.3% of patients and Notch4 in 68.3% [15]. However, there is little known about the actions or the mechanisms of Notch signaling in HCCs [9, 13].

Growing evidence shows that Notch signaling may play an oncogenic role in HCC progression, by regulating certain signaling pathways and their associated genes, such as p27, p21 and myc [9]. In our previous study, the nuclear receptor gene NR4A2 was found to be involved in Notch-mediated downstream signaling pathways in cervical cancer cells [11]. NR4A2 belongs to the NR4A subfamily of the nuclear receptor family of transcription factors [18]. These NR4A family members affect multiple cell functions such as cell cycling, cell apoptosis and proliferation [19–21]. They also were found related to the progression of cancers such as cervical cancer, melanoma, bladder cancer, prostate cancer and lung cancer [18–20, 22]. However, their roles in carcinogenesis are poorly defined. We have demonstrated that NR4A2 might act as an oncogene in cervical cancer cells [11]. Presently, we will investigate further the effects of Notch signaling and NR4A2 in HCC cells.

Our preliminary studies showed that suppression of Notch signaling could result in HCC cell proliferation suppression, while the activation of NR4A2 increased cell proliferation. In the present study, we investigated further to understand the impacts of Notch signaling and NR4A2 on HCC cell growth and their correlation in HCC progression. We found that the activation of both Notch and NR4A2 stimulated HCC cell proliferation and anti-apoptosis. Knockdown of Notch/NR4A2 signaling could stop HCC cell growth. These new findings support the oncogenic functions of Notch genes and the NR4A2 gene and may provide potential and valuable approaches for the development of novel anti-HCC drugs via blocking the crosstalk of Notch and NR4A2.

## RESULTS

Notch signaling is activated highly in HCC tissues, predicting its oncogenic role [9, 15]. However, some other reports showed suppressive effects of Notch signaling in HCC cells [9, 12]. The controversy lead us to confirm the role of Notch signaling in HCCs. In this study, we investigated the expression of Notch1 in primary HCC tissues and the role of Notch signaling in HCC cells, along with the involvement of certain genes in Notch-mediated signaling networks. We hypothesized that Notch mediated downstream signaling in HCC cells via regulating the oncogenic NR4A2 gene.

### The expression of Notch1 and NR4A2 in HCC tissues

**Figure 1:**
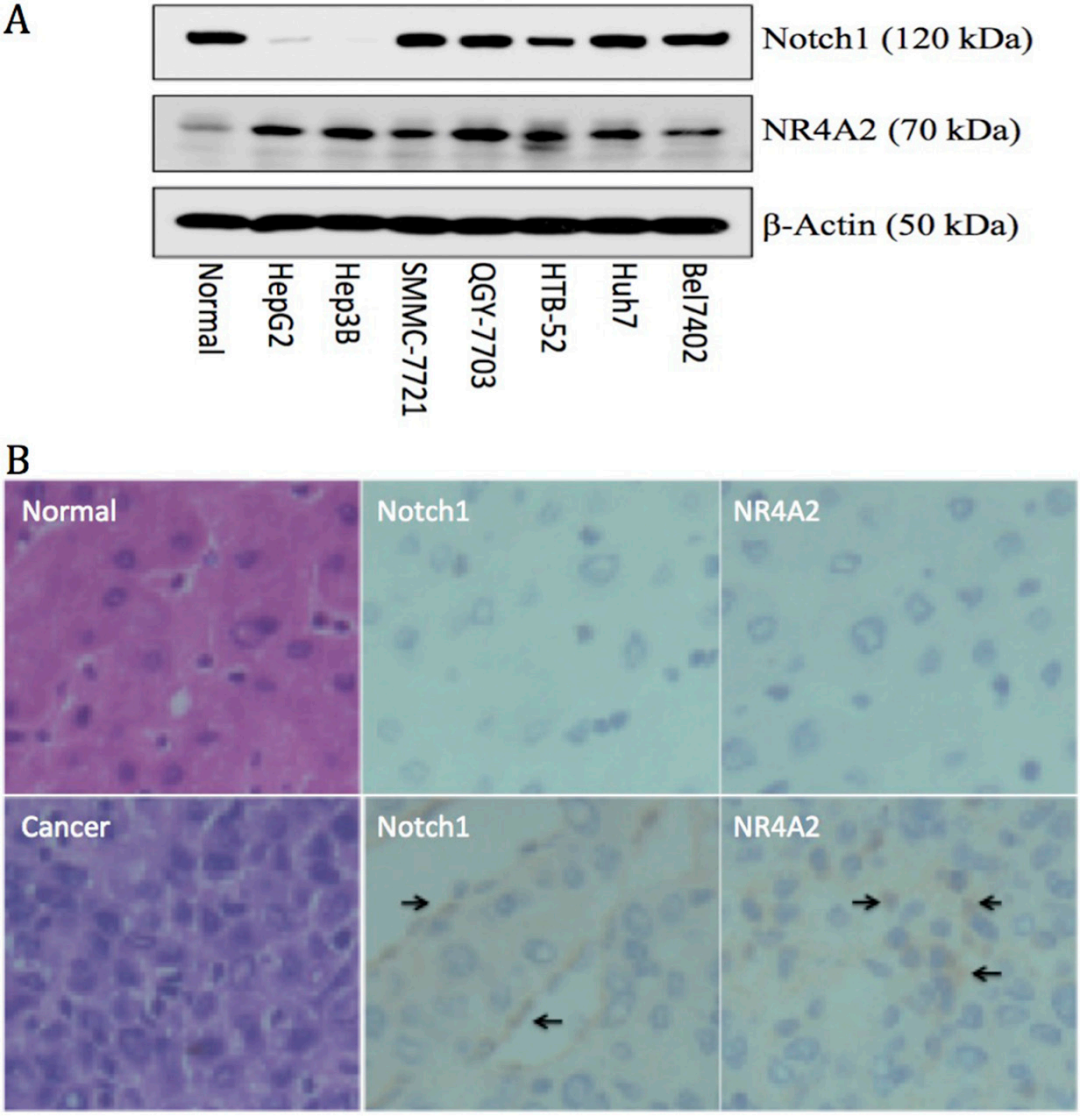
Up-regulation of Notch1 and NR4A2 in immortal HCC cell lines (**A**) and primary HCC tissues (**B**) in comparison with the normal controls. A. Western blot assay showed Notch1 was expressed highly in 5 out of 7 HCC cell lines, including Bel7402, Huh7, HTB-52, QGY-7721 and SMMC-7721, but not in Hep3B and HepG2, with NR4A2 being up-regulated in all 7 tested HCC cell lines. B. immuno-histochemistry assays confirmed the high expression of Notch1 and NR4A2 in primary HCC tissues with arrows pointing the positive expression, but not in normal tissues. The microscope images were shown at 40× magnification.

In order to better understand the correlation and interaction of Notch1 and NR4A2, we first investigated the expression of both genes in different HCC cells and in normal liver cells, primary HCC tissues and the adjacent normal liver tissues. Via western blot assay, we found that Notch1 is highly expressed in 5 out of 7 HCC cell lines (high in SMMC-7721, QGY-7703, HTB-52 (Sk-hep1), Huh7 and Bel7402 cells, but rare in Hep3B and HepG2 cells) and in normal liver cells. NR4A2 was expressed highly in all 7 HCC cell lines, but less in normal cells (Figure 1A). We further investigated the expression of Notch1 and NR4A2 in primary HCC tissues via qPCR and IHC assays. The qPCR assay showed that Notch1 at the mRNA level was increased highly in 56.2% of human HCC tissues (54 of 96 pairs) as compared to adjacent liver tissues, with NR4A2 being over-expressed in 46.8% (45 of 96) (data not shown). Similar results were observed at the protein level via the IHC assay (Figure 1B). These findings indicate that the activation of Notch and NR4A2 might be associated with HCC progression.

### The activation of Notch signaling induced cell proliferation

**Figure 2:**
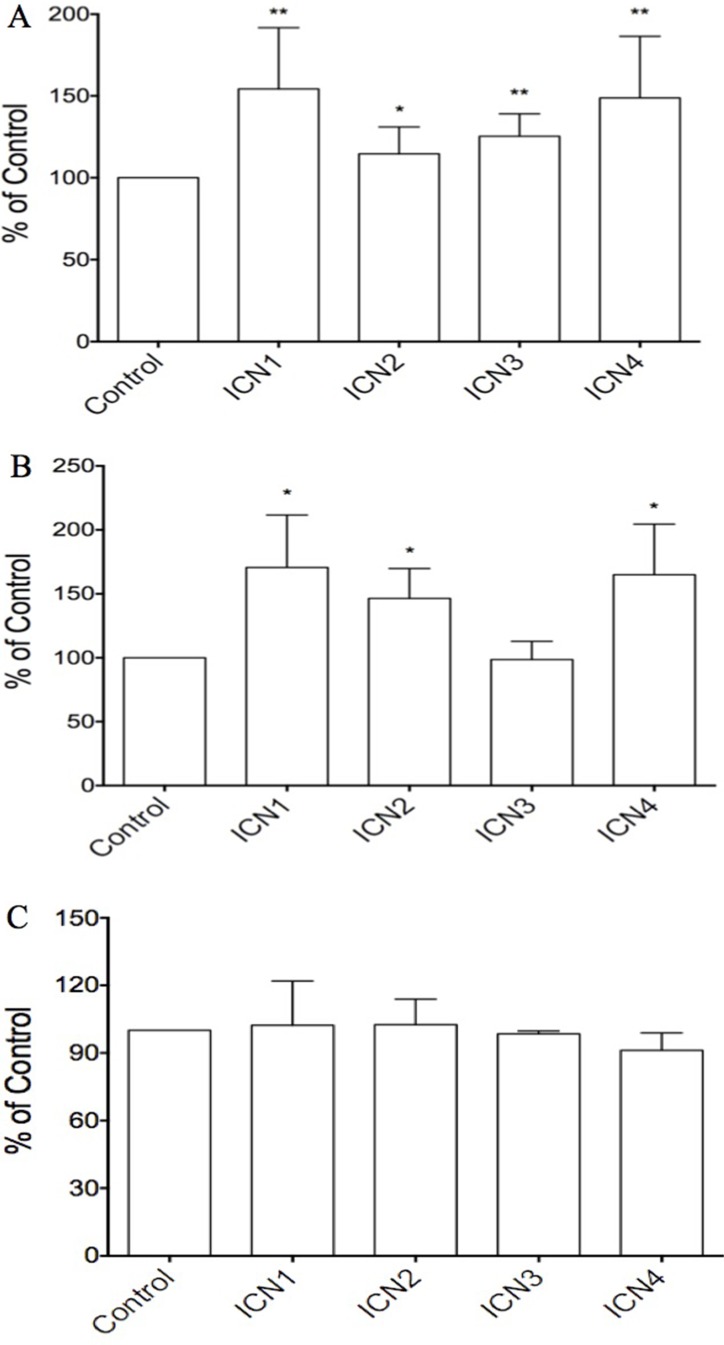
The effects of Notch signaling activation on cell proliferation in three HCC cell lines via over-expressing the Notch active forms ICN1, ICN2, ICN3 and ICN4 ICN1 and ICN4 could increase strongly cell growth in the HCC HTB-52 cells (**A**) and normal THLE2 cells (**B**), with ICN2 slightly increasing cell growth in both A and B. ICN3 increased cell growth in HTB-52 cells (A), but not in normal THLE2 cells (B). Interestingly, there was not significant effects observed in HCC HepG2 cells with over-expression of all four Notch active forms ICN1, ICN2, ICN3 and ICN4 (**C**). Statistical significance was shown with the asterisks (*) (**P < 0.01, *P < 0.05).

We hypothesized that Notch signaling may be a key oncogene in HCC progression. Three different cell lines including HCC HTB-52 cells, HCC HepG2 cells, and normal liver THLE2 cells, were chosen for the study. We found that Notch signaling activation, via transient transfection of the Notch active forms, enhanced cell proliferation in HCC HTB-52 cells and in normal liver THLE2 cells, but not in HCC HepG2 cells. As shown in Figure 2, the over-expression of the Notch active forms of all four Notch receptors (ICN1, 2, 3 and 4), via transient transfection in HCC HTB-52 cells, could increase cell proliferation. The increased rates are 54.3% (ICN1), 14.6% (ICN2), 25.4% (ICN3) and 48.8% (ICN4) (Figure 2A). Notch activation (ICN1, 2 and 4) also was observed to increase cell proliferation in normal THLE2 cells, but not so with ICN3. The increased rates were 70.6% (ICN1), 46.5% (ICN2), -1.4% (ICN3) and 65.0% (ICN4) (Figure 2B). However, interestingly, we found that the effects of Notch activation was very limited in HCC HepG2 cells, with the ICN-induced rates being 2.3% (ICN1), 2.5% (ICN2), −1.6% (ICN3) and −8.8% (ICN4), while *t*-test analysis showed no significant change (Figure 2C). These results support that Notch signaling may play different roles in different kinds of liver cells and in a notch subtype-dependent manner.

### Over-expression of ICN1 and ICN4 induced cell cycle progression

**Figure 3:**
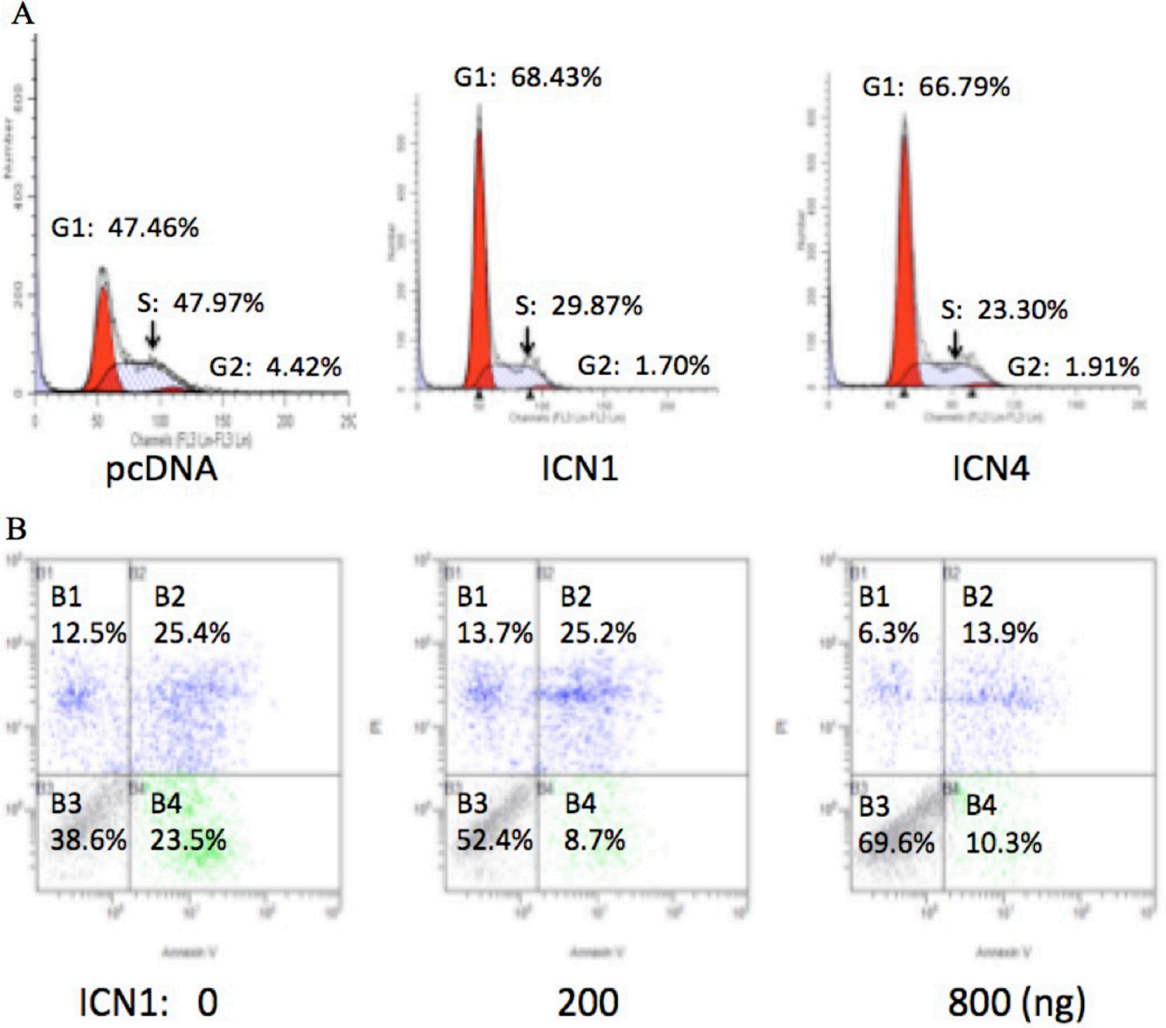
The effects of activated Notch signaling on cell cycle progression (**A**) and cell apoptosis (**B**) in HCC HTB-52 cells by FACS analysis. A. the over-expression of ICN1 and ICN4 induced cell cycle arrest in phase G1. B. ICN1 reduced cell apoptosis in a dose-dependent manner.

Notch signaling activation stimulated HCC cell growth as described above. We did further cell cycle assays on HCC HTB-52 cells and evaluated the effects of Notch signaling activation on cell cycle progression via over-expressing ICN1 and ICN4. The analysis showed that both ICN1 and ICN4 induced cell cycle arrest. As seen in Figure 3A, the percentages for the control group are 48% (Phase G1), 4% (G2) and 48% (S), with 68% (G1), 2% (G2) and 30% (S) in the ICN1 group, and 67% (G1), 2% (G2) and 31% (S) in the ICN4 group. A significant increase in the G1 phase was observed in the ICN1 and ICN4 groups in comparison to the control group.

### Over-expression of ICN1 decreased cell apoptosis

Our further apoptosis assays show that Notch1 activation via transient ICN1 transfection decreased HCC HTB-52 cell apoptosis compared to cell apoptosis resulting from that just using transfection agents. Cells were transfected using the transfection agent Lipo-2000, that results in some cell death, and were continuously cultured for 2 days without changing the medium. As shown in Figure 3B, the percentages of visible cells were 38.6% for the control, 52.4% for ICN1 (200 ng) and 69.6% for ICN1 (800 ng) while the apoptotic percentages of cells apoptosis (all apoptosis and necrosis together) were 61.6% for the control group, 47.6% for ICN1 (200 ng) and 30.4% for ICN1 (800 ng). This supports that Notch1 activation decreases cell apoptosis while increasing cell proliferation.

### The effects of Notch signaling activation on gene expression

**Figure 4:**
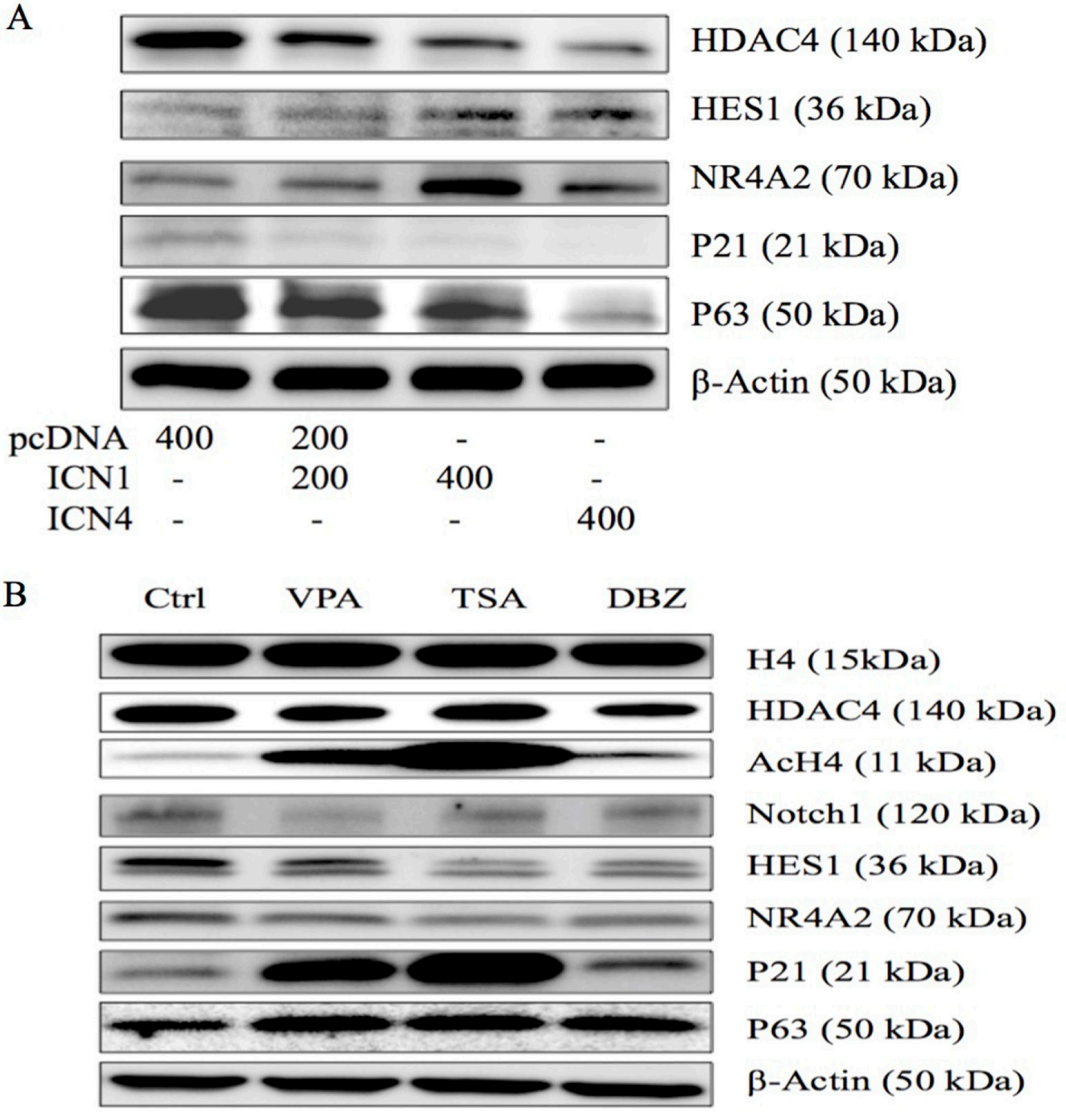
Western blot analysis was done to evaluate gene expression in HCC HTB-52 cells (**A**) The effects of Notch activation on certain genes (HDAC4, HES1, NR4A2, p21 and p63) via over-expressing ICN1 (200 ng, 400 ng) and ICN4 (400 ng). Activated Notch signaling decreased the expression of HDAC4, p21 and p63, and increased the expression of HES1 and NR4A2. (**B**) The three compounds, VPA, TSA, and DBZ, affected gene expression, with all three increasing the expression of AcH4, p21 and p63, and decreasing the expression of Notch1, HES1 and NR4A2, along with a slight decrease in HDAC4, but not in H4.

In our previous study, certain signaling pathways have been shown to be involved in cell growth arrest mediated by Notch1 signaling activation. We also observed the effects of Notch1 on certain genes in cervical cancer Hela cells [11]. NR4A2, as well as VPA, modulated the expression of these genes in HCC HTB-52 cells [9]. Thus, we investigated the effects of Notch activation on NR4A2 and certain other genes. As seen in Figure 4A, western blot analysis shows that Notch activation (ICN1 and ICN4) in HCC cells increased the expression of the Notch target gene HES1 and the nuclear receptor NR4A2 (Nurr1), but suppressed the expression of HDAC4 and tumor suppressors p21 and p63, indicating the involvement of NR4A2 and tumor suppressors in Notch-mediated signaling cascades.

### The effects of NR4A2 on cell growth via acting as an oncogene

**Figure 5:**
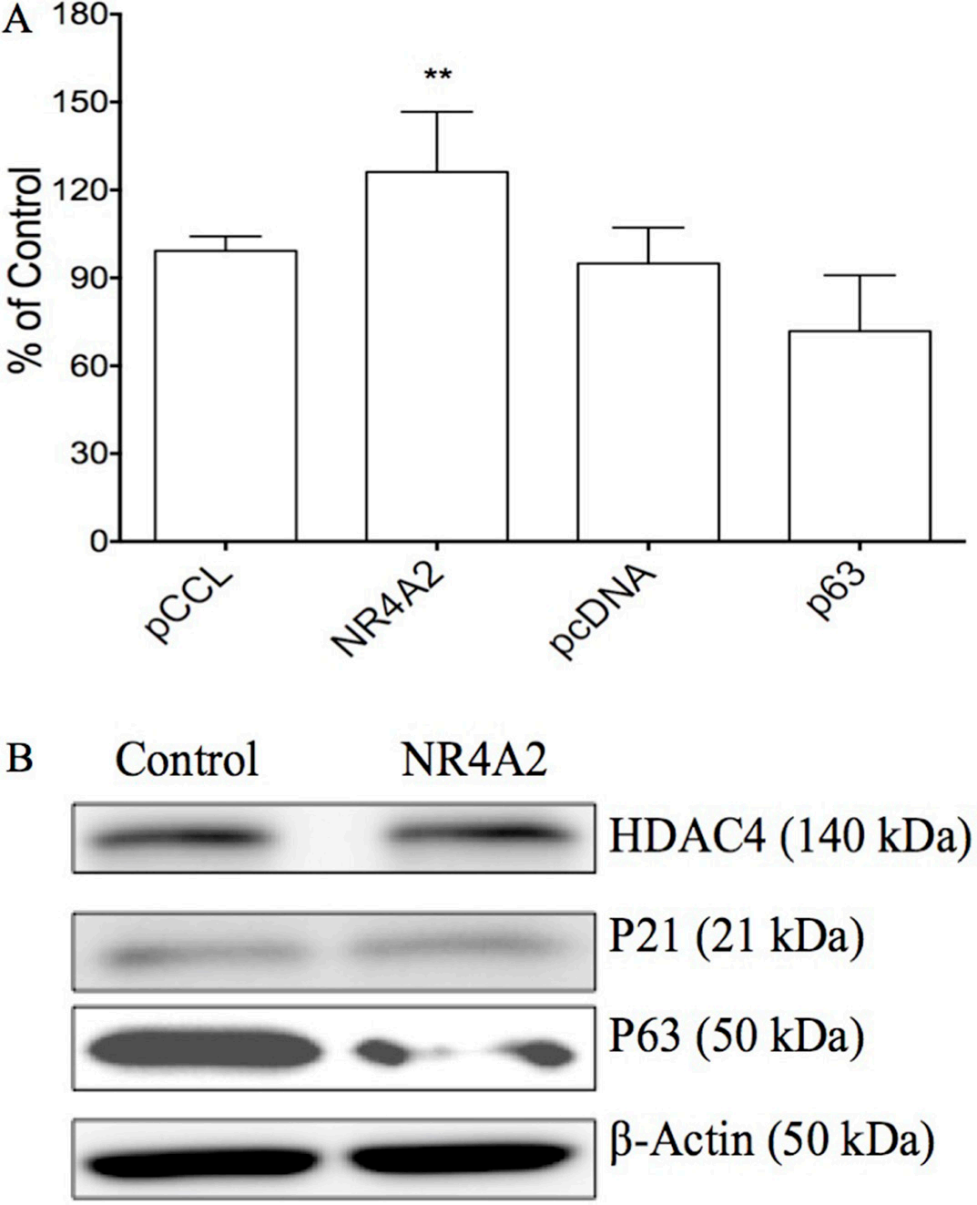
The effects of NR4A2 on cell proliferation and other gene expression in HTB-52 cells via over-expressing NR4A2 (**A**) cell proliferation assays show that NR4A2 over-expression stimulated cell growth, with an increased rate of 27%. The tumor suppressor p63 suppressed cell growth with a decrease of 23%. Statistical significance was shown with the asterisks (**P < 0.01). (**B**) Western blot analysis show that the over-expression of NR4A2 suppressed the expression of p63, but did little to HDAC4 and p21.

Our previous study showed the effects of Notch1 on certain genes such as NR4A2, p63 in cervical cancer Hela cells [11]. We further evaluated the effects of these genes on cell growth in HTB-52 cells. The plasmids carrying the gene NR4A2 or p63 were transiently transfected in HTB-52 cells and analyzed for their effects on cell proliferation. The assay identified that over-expression of NR4A2 induced HCC HTB-52 cell proliferation, with an increased rate of 26%, while tumor suppressor p63 induced suppression with a decreasing rate of 23% (Figure 5A). Further assays at the protein level by western blot showed that NR4A2 induced a decrease in the tumor suppressor p63, with no or little effects on p21 and HDAC4 (Figure 5B), indicating that NR4A2 may act particularly as a p63 negative regulator in HCC cell growth.

### The effects of Notch regulators on HCC cell growth

**Figure 6:**
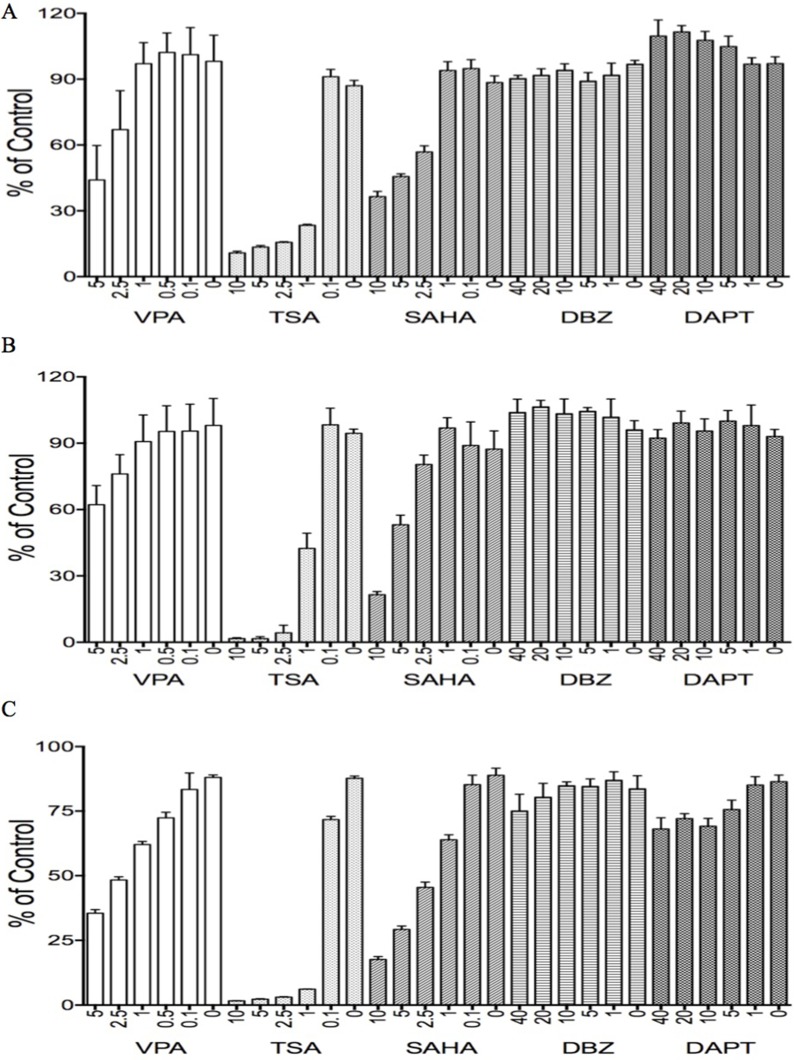
The effects of various tested compounds on cell proliferation The two HDAC inhibitors, TSA and SAHA, significantly suppressed cell growth in all three types of HCC cells, HTB-52 (**A**), HCC HepG2 (**B**) and liver cells THLE2 (**C**). The dual HDAC/Notch regulator, VPA, partly suppressed cell growth in all three cell lines as well. One-way ANOVA analysis showed the significant suppressive effects with TSA and SAHA in each cell line, as well with the effective trends in the treatments of VPA among the serial different concentrations. However, the significant effects of the Notch inhibitors DBZ and DAPT were not observed in these cells.

Our previous studies identified that the HDAC inhibitor VPA suppressed tumor cell growth and stimulated Notch signaling in some tumor cells such as cervical cancer cells via acting as a Notch stimulator [11, 24]. Thus, we hypothesized that VPA might affect HCC cells via modulating Notch signaling. We evaluated the effects of VPA on HCC HTB-52, HepG2 cells, and normal liver THLE2 cells, along with other Notch inhibitors such as DBZ, DAPT, and other HDAC inhibitors such as TSA and SAHA, done at their customary concentrations (Figure 6).

VPA displayed significant suppression in HCC and normal liver THLE2 cells in a dose-dependent manner, with the suppressive rates using VPA at 5 mM being 57% in HTB-52 cells (Figure 6A), 35% in HepG2 cells (Figure 6B) and 65% in THLE2 cells (Figure 6C). The two HDAC inhibitors TSA and SAHA showed more potent inhibitory activity in all HCC cells and in normal liver cells. In comparison with that, the two Notch inhibitors DBZ and DAPT had no effect in these cells (Figure 6).

### The effects of Notch regulators on cell cycle progression and apoptosis

We further evaluated the effects of these factors at their customary concentrations on cell cycle progression and cell apoptosis. For cell cycle arrest, FACS assays were done in the same three cell lines HTB-52, HepG2 and THLE2. In HCC HTB-52 cells, VPA, TSA and SAHA induced growth arrest at phase G2, with the rates being 13.7% (VPA: 4 mM), 12.3% (TSA: 1 μM) and 8.8% (SAHA: 1 μM), compared to 4% for the control. There was little change for DAPT at 50 μM (6%) and no change with DBZ at 50 μM (4.8%) (Table 1). In HCC HepG2 cells, these factors slightly induced a growth arrest at phase S, with a slight increase of the rates being 15% (VPA: 4 mM), 12.4% (TSA: 1 μM), 13.7% (SAHA: 1 μM), 12.7% (DBZ: 50 μM) and 14.1% (DAPT: 50 μM), compared to 10.3% for the control (Table 1). However, in normal liver THLE2 cells, these factors slightly induced a growth arrest at phase G, with the rates being 49.7% (VPA: 4 mM), 47.5% (TSA: 1 μM), 49.3% (SAHA: 1 μM), 47.3% (DBZ: 50 μM), and 47.4% (DAPT: 50 μM) compared to 45.5% for the controls (Table 1). We further confirmed that the two compounds, VPA and TSA, induced HCC HTB-52 cell apoptosis, with the percentage of apoptosis and necro-apoptosis being 89% (VPA) and 90% (TSA), but DBZ did not (data not shown).

**Table 1:** The effects of different agents on HCC cell cycle progression

Agents	Cell types	Phases			Changes
		G1	G2	S	
Control	HTB-52	80.32 ± 1.94	3.97 ± 1.62	15.71 ± 2.31	
	HepG2	78.04 ± 1.34	11.17 ± 3.08	10.33 ± 2.07	
	THLE2	45.51 ± 1.36	29.48 ± 3.37	24.80 ± 4.43	
DMSO	HTB-52	77.33 ± 3.36	5.43 ± 0.20	17.24 ± 3.33	-
	HepG2	78.07 ± 3.01	10.61 ± 2.03	10.44 ± 2.01	-
	THLE2	45.80 ± 1.18	28.46 ± 1.93	25.74 ± 2.96	-
VPA	HTB-52	80.59 ± 2.87	**13.73 ± 0.64**	5.68 ± 2.23	G2
	HepG2	79.79 ± 4.94	10.98 ± 2.35	**14.95 ± 3.33**	S
	THLE2	**49.65 ± 1.04**	31.71 ± 2.11	18.64 ± 1.08	G1
TSA	HTB-52	78.54 ± 1.92	**12.33 ± 0.10**	9.13 ± 1.90	G2
	HepG2	75.57 ± 0.90	11.54 ± 2.92	**12.41 ± 4.10**	S
	THLE2	**47.45 ± 2.17**	34.66 ± 3.10	17.89 ± 1.61	G1
SAHA	HTB-52	84.77 ± 5.20	**8.80 ± 2.50**	6.43 ± 0.94	G2
	HepG2	68.62 ± 3.97	10.97 ± 1.95	**13.68 ± 4.80**	S
	THLE2	**49.27 ± 2.02**	30.24 ± 3.03	20.49 ± 3.27	G1
DBZ	HTB-52	78.28 ± 1.41	4.82 ± 0.90	16.90 ± 1.83	-
	HepG2	73.36 ± 1.74	10.77 ± 2.11	**12.66 ± 4.79**	S
	THLE2	**47.26 ± 1.32**	29.25 ± 1.61	23.47 ± 2.61	G1
DAPT	HTB-52	77.65 ± 0.73	**6.00 ± 0.37**	16.35 ± 1.10	G2
	HepG2	74.66 ± 1.30	10.84 ± 2.92	**14.09 ± 3.69**	S
	THLE2	**47.35 ± 1.14**	28.97 ± 2.32	23.67 ± 1.88	G1

### The effects of Notch regulators on HCC cell morphological change

**Figure 7:**
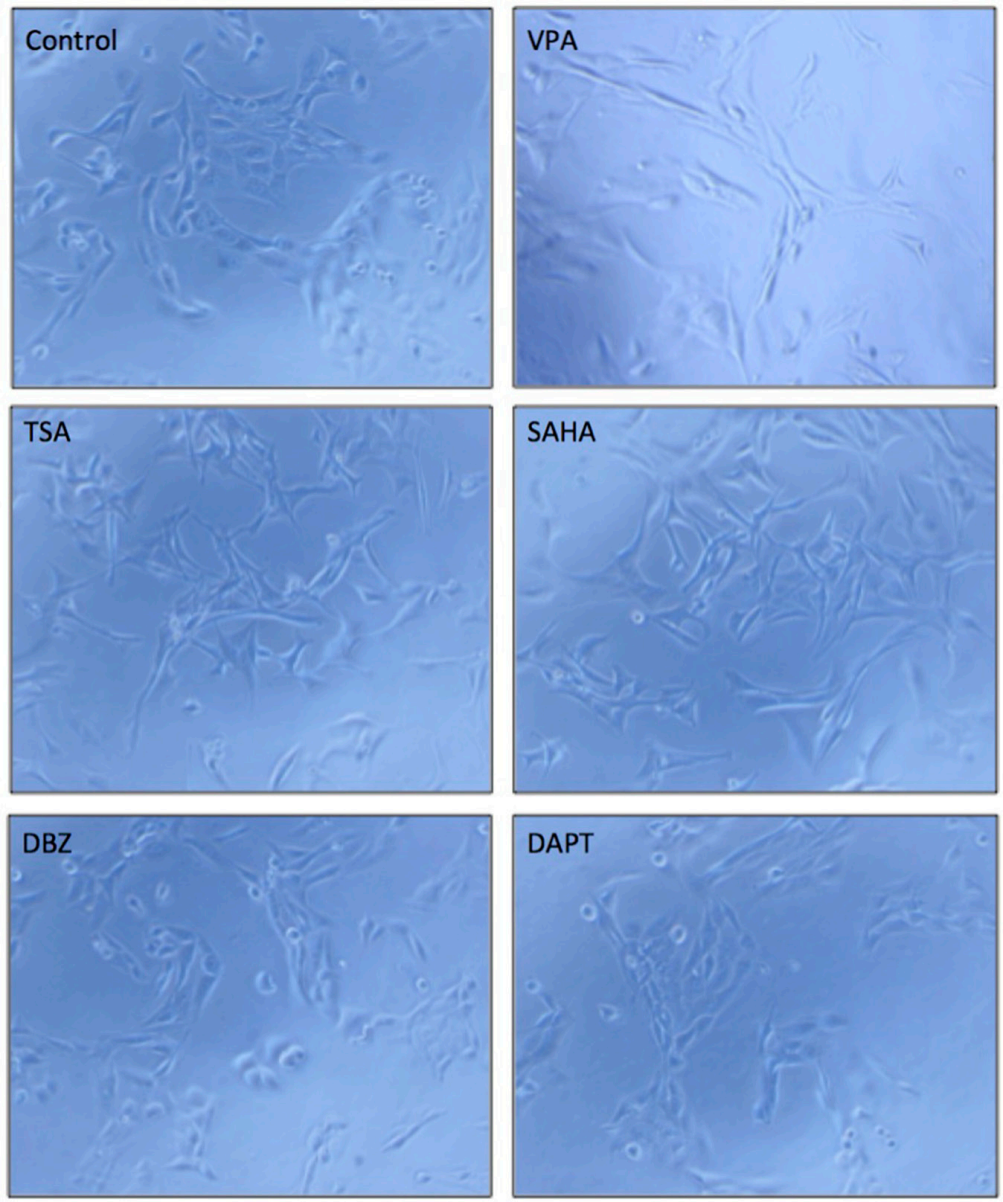
The effects of the tested compounds on cell morphological change in HTB-52 cells A VPA, TSA and SAHA obviously induced cell morphological change, while DBZ and DAPT did not significantly. The microscope images were shown at 10× magnification.

Cell morphological change is strongly related to cell differentiation and tumor metastasis [25]. VPA was reported to affect cell differentiation in many cancer cells [26, 27]. We tested the effects of VPA and five other compounds in HCC HTB-52 cells. The three compounds, VPA, TSA and SAHA, were observed to induce HCC HTB-52 cell morphological change, but DBZ and DAPT did not (Figure 7). We also analyzed the effects of VPA on the expression of the metastasis-related genes such as MMP2, Twist and Snail. We found that MMP2 was increased, with Twist decreased and Snail not changed (data not shown), supporting that Notch regulators might possibly be options for the development of anti-HCC drugs.

### The effects of Notch regulators on Notch1 and NR4A2

As discussed above, both Notch1 and NR4A2 could stimulate HCC cell proliferation and inhibit cell apoptosis, supporting their oncogenic functions in HCC cells. Therefore, we further investigated the effects of VPA, along with the other two molecules, TSA and DBZ, on histone acetylation, Notch and other associated genes, particularly NR4A2. We found that all three molecules increased the expression of AcH4, but not H4 and HDAC4. They also increased the expression of tumor suppressor p21 and p63. These molecules decreased the expression of Notch1, Notch target gene HES1and NR4A2 (Figure 4B). These molecules affected HCC cell growth possibly via regulating histone acetylation and Notch downstream signaling, but they might modulate different signaling pathways.

### Knockdown of Notch signaling suppressed NR4A2 and reversed ICN1-induced cell proliferation

**Figure 8:**
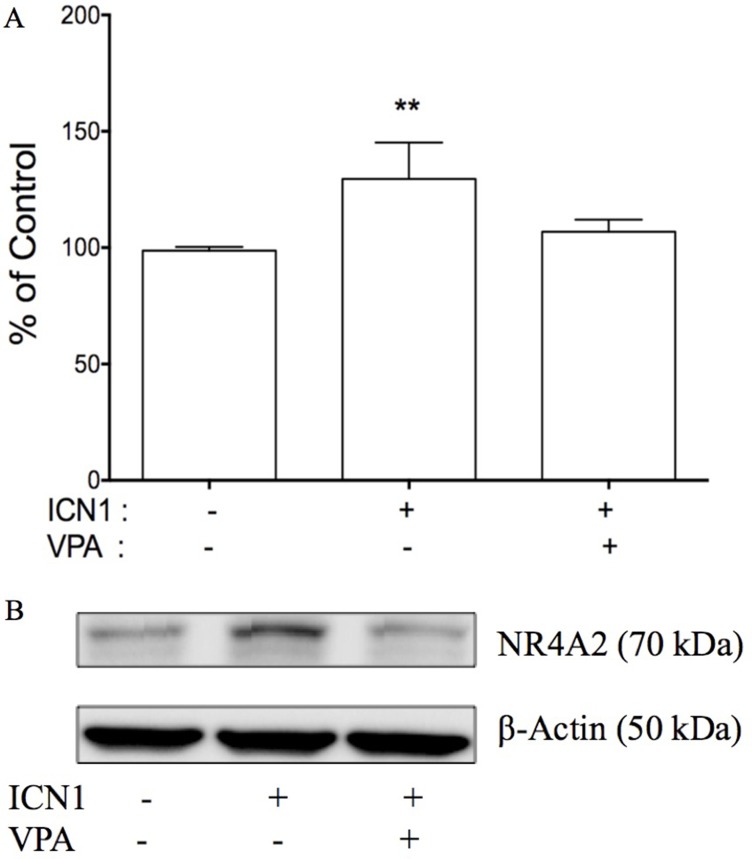
The effects of Notch signaling on cell proliferation and NR4A2 in HTB-52 cells (**A**) the over-expression of ICN1 increased HTB-52 cell proliferation, but the Notch inhibitor VPA reversed an ICN1-induced increase. Statistical significance was shown with the asterisks (**P < 0.01). (**B**) ICN1 induced an increase in NR4A2, but VPA erased the ICN1-induced increase of NR4A2.

By understanding that the activation of Notch signaling increased HCC cell growth, its inactivation decreased growth, and with the involvement of genes such as NR4A2, p21 and p63, we thus hypothesized that NR4A2 functioned as an oncogene via directly involving Notch-mediated HCC carcinogenesis and the downstream signaling networks. In the present study, we tested to see if the negative Notch regulator VPA could reverse the effects of Notch signaling in HCC cells. The over-expression of the Notch1 active form ICN1 stimulated HCC HTB-52 cell growth, but VPA could erase ICN1-induced increase in cell growth (Figure 8A). As seen in Figure 8A, ICN1 induced an increase (29.6%). However, VPA reduced the ICN1-induced increase to 6.8%, compared to that of the control group. Furthermore, western blot analysis showed that VPA could erase Notch-induced gene expression. Shown in Figure 8B, VPA reversed an ICN1-induced increase of NR4A2. These support that NR4A2 plays an oncogenic role in HCC cells, with VPA acting as a negative Notch/NR4A2 signaling regulator.

## DISCUSSION

Hepatocellular carcinoma (HCC) is the most prevalent primary liver cancer and one of the most malignant cancers [9, 28]. Conventional treatments have limited anti-HCC efficacy and easily induce severe toxic side-effects. Thus, scientists pay more attention to the development of more effective anti-HCC targeted drugs having less toxicity. One challenge is that the molecular mechanisms of HCC carcinogenesis are unclear. Various signaling pathways and associated genes have been demonstrated to be involved in HCC progression [4, 9]. One of them is the highly conserved Notch signaling that is extremely critical in development and cell control [10, 29]. This signaling has been found involved widely in the carcinogenesis process [9]. Our current studies support that Notch signaling activation stimulates HCC cell proliferation. Our previous studies identified the multiple functions of Notch signaling in different types of cancers, and supported that Notch signaling was more diversified and complicated than expected [9, 11]. Thus, cancer type-specific strategies should be carefully considered when developing anti-cancer drugs against Notch signaling.

The functions and the precise molecular mechanisms of Notch signaling are not fully clear in HCCs, and are even controversial in different independent studies [9, 13]. However, accumulating evidence supports that Notch signaling may be a HCC diagnostic marker and potentially be an anti-HCC drug target [30–32]. The high signature of Notch signaling has been observed in many HCC tissues [15, 16, 30]. We compared the difference of Notch1 existing in primary HCC tissues and in adjacent liver tissues and found that Notch1 is up-regulated highly in over 50% of tumor tissues and most tested HCC cell lines, identical to other reports [16, 31]. Further assays display that Notch signaling activation via over-expressing Notch active form (ICN) could stimulate growth of HCC HTB-52 and normal liver THLE2 cells with a high Notch signature. Moreover, we found that Notch signaling worked in a Notch subtype-dependent manner, with ICN1 and ICN4 displaying more effects in both cell lines. Notch signaling knockdown using Notch inhibitors suppressed HCC HTB-52 cell growth. We did not observe a significant effect of Notch activation on HCC HepG2 cells with low Notch signature. Thus, the strategy of developing anti-HCC drugs via targeting Notch signaling needs to be carefully evaluated [9, 13].

The evidence implicates the impacts of the nuclear receptor NR4A family on cancer progression, although their roles in HCCs are unknown [18, 33]. Our previous study also showed that NR4A2 stimulated cervical cancer cell growth [11]. Presently, we found that NR4A2 was up-regulated in many primary HCC tissues and immortal HCC cell lines, and co-existed with Notch1. Also, NR4A2 induced an increase in HCC cell growth just as Notch signaling did. Further investigations at the molecular level found out that Notch activation/inactivation modulated the expression of NR4A2. Both Notch1 and NR4A2 negatively co-regulated tumor suppressor p63. NR4A2 also was recently reported to be involved in the p53-mediated signaling networks [34]. These findings support a correlation between Notch1 and NR4A2, both of which may affect positively HCC progression via co-regulating downstream signaling pathways, particularly by suppressing tumor suppressors. This new discovery may provide a potential anti-HCC approach via blocking the Notch/NR4A2 signaling cascade.

Interventions of Notch-mediated signaling have been applied to treating various cancers [11, 24, 35]. In our previous study and in other reports, the HDAC inhibitor VPA has been demonstrated able to act as a positive/negative Notch regulator in a cancer type-dependent manner [9, 23]. We confirmed the oncogenic effects of Notch signaling in HCC tissues and cells. Thus, we tested VPA and other HDAC/Notch inhibitors for their effects in HCC cells and their involvement in Notch/NR4A2-mediated signaling pathways. Both TSA and SAHA displayed the most potent anti-HCC efficacy. VPA also suppressed cell growth, similar to our previous reports [24]. But the Notch inhibitors DBZ and DAPT did not show significant effects on HCC cell growth. These compounds are different in their precise mechanisms and functions in HCC treatments although all of them induced acetylation of histone 4, decreased Notch1 and NR4A2, and increased the tumor suppressors p21 and p63. Particularly, VPA is widely used to treat many cancers as an adjuvant in combination with various anti-cancer agents [36, 37]. In the present study, VPA could suppress HCC cell growth and block Notch/NR4A2-mediated signaling. Furthermore, VPA could erase the Notch1-induced increase in HCC cell growth via directly knocking down Notch1-induced up-regulation of NR4A2, supporting that VPA in combination with others is potentially an anti-HCC drug by targeting the Notch/NR4A2 signaling pathway [9, 38, 39]. Further combination therapeutics are currently under investigation.

Conclusively, we demonstrated that the nuclear receptor NR4A2, besides Notch1, was highly expressed in HCC tissues and HCC cells. These two genes also play oncogenic roles in HCC cells and may serve as predictive HCC oncogenic markers. Knockdown of these genes could slow down HCC cell growth. Thus, intervention of Notch1/NR4A2-mediated signaling may provide a promising opportunity for the development of HCC-targeted drugs. VPA as an adjuvant agent may be an option for a combination treatment in HCC patients.

## MATERIALS AND METHODS

### Materials

Valproic acid sodium salt (VPA), DAPT (LY-374973, N-[N-(3,5-Difluorophenacetyl)-L-alanyl]-S-phenylglycine t-butyl ester), dibenzazepine (DBZ) and trichostatin A (TSA) and suberoylanilidehydroxamic acid (SAHA), were purchased from Sigma (St. Louis, MO). Antibodies of p21 (sc-756), p63 (sc-8343), COX2 (sc-7951), histone 4 (H4, sc-10810), HDAC4 (sc-11418), acetylation of histone 4 (AcH4, sc-8660-R), Notch1 (sc-23299), NR4A2 (Nurr1, N-20, sc-991), HES1 (sc-25392) and β-actin (sc-1616-HRP) were purchased from Santa Cruz Biotechnology (Santa Cruz, CA). The plasmids expressing the intracellular domains of the four Notch receptors (ICN1, ICN2, ICN3, and ICN4) were gifts from Dr. Wu (University of Florida). The plasmids with the genes NR4A2 (Nurr1) and p63 were obtained from Addgene (www.addgene.org), including pcDNA-p63 (27008), pCCL-NR4A2 (plasmid 35000) and the control pCCL (10881).

### Tumor specimens

Fresh surgical specimens, including tumor tissues and adjacent non-tumor liver tissues, were obtained from 96 HCC patients at the Qidong Liver Cancer Institute (Jiangsu, China), with the protocol being approved by the Jiangsu Medical Ethics Committee. All tissue samples were immediately frozen in liquid nitrogen after surgery and stored at −80°C before further analysis.

### Cell culture and transfection

Human cell lines HepG2, Hep3B, Huh7, HTB-52 (Sk-hep-1) and THLE2 were purchased from ATCC (American Type Culture Collection, Manassas, VA). Human HCC cell lines SMMC-7721, QGY- 7703, and BEL-7402 were obtained from the Cell Bank of the Chinese Academy of Sciences (Shanghai, China). All cells were maintained in DMEM/MEM medium supplemented with 10% fetal bovine serum (FBS) and 1% penicillin/streptomycin, with incubation at 37^°^C in a 5% CO_2_ atmosphere.

For cell transfection, 500 μl of cells (2 × 10^5^ cells/ml) were plated in each well of a 24-well plate. Two μl of Lipofectamine^TM^ 2000 (Lipo-2000) and 0.8 μg of DNA were added separately in each vial with 50 μl Opti-MEM transfection medium, and combined together after 5–10 minute incubation. The DNA/Lipo2000 complexes were incubated continuously for 20–30 minutes and then added to each well. The transfected cells were incubated for 48 hours and assayed for cell cycling and apoptosis, or alternatively, in a cell proliferation assay, growth medium was changed 4–5 hours later and cells were further incubated for 72-hours. The experiments were done separately in triplicate.

### RT-PCR and real-time PCR

Total RNA was extracted from tissues and cells with Trizol reagent (Invitrogen). RT-PCR was performed as described in the protocols (Invitrogen, Carlsbad, CA). The primers and conditions for RT-PCR analyses were as previously described (23), with the exception of NR4A2 (Nurr1) primers (Forward: 5′-AGA GAC GCG GAG AAC TCC TA-3′, Reverse: 5′-AGG CAT GGC TTC AGC CGA GT-3′). PCR was regularly amplified for 35 cycles, more or less, due to the difference in RNA abundance of these investigated genes. Primers for real-time PCR analyses were the same as described above. Real-time PCR was performed by using SYBR Green PCR master mix from QuantStudio™ 7 (Life technologies, USA). Properly diluted cDNA was used in a 10 μl qRT-PCR, in triplicate for each gene. β-actin was used as the internal control and results were calculated by applying 2^−ΔΔCT^ methods. The experiments were done separately in triplicate.

### Western blot analysis

Western blot was employed as described in the protocol (Santa Cruz). Briefly, cells were harvested, re-suspended in RIPA buffer with protein cocktail inhibitors, homogenized with a 21-gauge needle, mixed with the loading buffer and heated for 5 min at 95°C. Supernatants were loaded to run on 8–16% Tris-glycine gel after centrifugation at 10,000 × g. Protein was transferred onto a nitrocellulose membrane (Millipore, Bedford, MA, USA). The membranes were blocked with 5% fat-free milk and 0.1% Tween-20, washed and incubated with primary antibody. The membrane was washed again and incubated with second antibody (Santa Cruz). Films were developed according to the ECL system protocol (Amersham Biosciences, England). The experiments were done separately in triplicate.

### Cell apoptosis and cell cycle analysis

Cells were plated on 6-well plates and incubated overnight. The test compounds were added. Cells were incubated again overnight and then were harvested for the assay. For the apoptosis assay, the Annexin V-FITC apoptosis detection kit (Cat. No.: APOAF) was purchased from Sigma (Saint Louis, MO). Into 500 μls of cell suspension was added 5 μl of Annexin V-FITC conjugate and 10 μl of a propidium iodide solution. The mixture was incubated for 10 minutes and assayed to determine fluorescence using a Beckman-Coulter Gallios analyzer. Data were analyzed with Gallios software. For the cell cycle assay, the Coulter DNA Prep reagents kit (Cat. No.: PN 6607055) from Beckman Coulter (Fullerton, CA) was used. Analysis was done on a Beckman-Coulter Epics FC500 analyzer using CXP software for acquisition and the ModFit LT v3.1 (Verity Software) for cell cycle modeling. Both experiments were repeated three times.

### Cell proliferation assay

The cell proliferation assay (Promega, Madison, WI) was performed as described previously (23). Briefly, 50 μl aliquots of medium with different concentrations of compounds were added to 96-well plates. All compound concentrations were tested in triplicate. Another 50 μl of the cell stock (1 × 10^5^ cells/μl of media) was dispensed into each well and the plates were incubated at 37°C in a CO_2_ incubator for 3 days. Following the incubation period, 15 ml of the dye solution was added to each well and the plates were then incubated at 37°C for 4 hours, followed by the addition of 100 μl per well of the solubilization solution. The plates were incubated at 37°C until the contents in each well became a uniform-colored solution. The absorbance was measured and recorded at 570 nm by a Victor Plate Reader (PerkinElmer, Boston, MA).

### Immunohistochemistry (IHC) analysis

For IHC staining, paraffin-embedded tissues were sectioned, mounted on positively charged glass slides (Thermo Scientific, MA, USA), baked, deparaffinized and rehydrated. Antigen retrieval was completed by heating slides in citrate buffer (10 mmol/L; pH 6.0) for 20 min. Sections were incubated overnight at 4°C with mouse monoclonal antibody Notch1 (1:300) or rabbit polyclonal antibody NR4A2 (Nurr1) (1:200) and then washed with PBS. The washed sections were incubated respectively with HRP-conjugated goat anti-rabbit IgG secondary antibodies and goat anti-mouse IgG secondary antibodies (EarthOx, CA, USA) at room temperature for 30 min. and visualized with an Eclipse Ci-s (Nikon, Japan).
